# Analysis of Deformation and Prediction of Cracks in the Cogging Process for Die Steel at Elevated Temperatures

**DOI:** 10.3390/ma13245589

**Published:** 2020-12-08

**Authors:** Marcin Kukuryk

**Affiliations:** Faculty of Mechanical Engineering and Computer Science, Czestochowa University of Technology, 42-200 Czestochowa, Poland; kukurykm@itm.pcz.pl

**Keywords:** cogging process, FEM, strain, stress, damage, shaped anvils

## Abstract

In this paper, an analysis of a three-dimensional state of strain and stress in the case of the hot cogging process of X32CrMoV12-28 die steel with the application of the finite element method is presented. The results of the investigations connected with the simulation of the kinematics of metal flow and thermal phenomena are presented, accompanied by prognosing the formation of ductile fractures in the course of the hot cogging process conducted with the application of three different shape tools and of a proposed deformation criterion of the loss of cohesion. The applied anvils were found to be highly effective in the aspects of distribution of effective strains and stresses, absence of tensile stresses in the axial zones of a forging, and also of a significant thermal stability in the internal layers of a deformed material. The developed course of changes in the deformation of the damage factor in the case of forging in the investigated anvils renders it possible to predict the situation and the phase of deformation in which the loss of cohesion by a deformed material will occur. The comparison between the predicted and the experimental results showed a good agreement.

## 1. Introduction

The natural non-uniformity of a structure and the defects of an initial billet, as well as the non-uniformity of deformations resulting from the technological cogging process, contribute to different final properties of forgings. This multi-faceted and complicated influence is connected with changes observed in the course of hot deformation space stresses, strains and the temperature of a material [1,2,3,4,5]. In order to meet these extremely significant challenges, modification of the technology of forging alloy steels, and that of high-alloy ones, by means of plastic deformations ought to be conducted with the application of appropriate forging tools and the best possible technological parameters [6,7,8,9]. The issue of selection of an appropriate technological forging process for obtaining forgings which would have high levels of mechanical properties, and be free of internal defects, constitutes an extremely significant problem [10,11,12,13]. From the point of view of technology, what is particularly significant is the state of strain and stress in the axial zone of a forged material because it is in this area of an ingot that the largest clusters of the following types of defects are found: micro-shrinkages, discontinuities and internal cracks, which, in the course of the technological process, ought to be noticeably reduced or completely eliminated [14,15,16]. Meeting qualitative objectives in the course of the cogging of alloy steels, and of high-alloy ones, is possible exclusively in conditions ensuring a favorable thermal-mechanical state, a feature of which is the presence, in a deformation valley, of compression stresses, in the conditions of the greatest restricting of the zones of tensile stresses [17,18,19,20]. Metal flow, the continuity of an internal structure and the structure and properties of a forged material are determined by the properties of the initial billet and, within the scope of forging technologies, are dependent upon the shape and dimension of forging tools, and also upon applied technological parameters [21,22,23,24].

In the course of the forging process, a material is formed into an appropriate shape; required mechanical properties, closely connected with the distribution of stresses and strains, are also obtained. Knowledge of these properties renders it possible to determine the areas with the highest plastic deformations and the situation of presumable cracks in a material. Analysis of the components of the states of strain and stress, and also of their changes in the course of the plastic shaping process, renders it possible to prognose maintaining a reforged material depending on the history of strains, owing to which one may initially prognose the internal quality of a forging at the subsequent stages of forging [25,26,27].

Determining the threshold workability of a material in the forging process is a significant issue from the point of view of designing the best possible technologies. One of the directions of investigations into this question was an attempt to determine the properties of a material and the parameters of the cogging process which result in the disturbance of the cohesion of the material and in the formation of cracks. The results of the conducted investigations demonstrated the significant influence exerted by the state of stress, strain rates, temperature, the parameters of microstructure and also by the conditions of friction on the contact surface between a material and a tool upon the threshold value of effective strain, after exceeding which a material cracks [28,29].

As a result of investigations into this issue, a number of criteria relevant to the formation of cracks using different analytical relationships based upon the values of stresses, integrated by means of material strain, were developed [30,31,32]. Nevertheless, comparative analysis of the results of different failure criteria results in formulating essentially different conclusions. The developed criteria do not render it possible to predict threshold strains in processes observed at changeable states of stress. In addition to that, numerous crack criteria are relevant to cold plastic processing, in the condition of the absence of the methodology of conducting hot deformation. In the condition of hot plastic deformation, it is not possible, unlike in an ambient temperature, to stabilize the temperatures of a deformed sample by means of applying a cooling medium. At significant strain rates, there may occur a significant increase in the temperature of a sample, brought about by the conversion of plastic deformation work into heat. A clear majority of the above-enumerated defects are not observed in the case of the modified strain criterion of the loss of cohesion proposed in this paper.

For that very reason, the original deformation criterion of the loss of cohesion, on the basis of the developed experimental curves of the threshold strains in the state of the function of the stress triaxiality and temperature, was applied. The possibility of predicting the situation and the phase of deformation in which the loss of cohesion of a plastically shaped material will occur is the source of numerous problems, but it is also indispensable for designing the best possible technological forging process appropriately.

The objective of this paper was to conduct an analysis of the distribution of effective strain, stresses and temperature and also to predict the formation of cracks in the course of plastic shaping in the three selected kinds of shaped anvils, complementary to one another, in the technological process of cogging X32CrMoV12-28 die steel.

## 2. Materials and Methods

### 2.1. The Basic Theories of the Thermal-Mechanical Finite Element Model

The finite element model of the cogging process is used. The solution is based upon a thermo-mechanical approach, combined with solving the Fourier equation for non-stationary heat flows. According to the variational principle, the basic equation for the finite element formulation can be written as follows [2,14]:

(1) $$\delta \Pi =\int_{V}\bar{\sigma }\delta \dot{\bar{\varepsilon }}dV+\int_{V}K{\dot{\varepsilon }}_{V}\delta {\dot{\varepsilon }}_{V}dV-\int_{S}F_{i}\delta u_{i}dS=0,$$

where $\delta {\dot{\varepsilon }}_{V}$ is the virtual volumetric strain rate, $\delta \dot{\bar{\varepsilon }}$ is the virtual effective strain rate, ${\dot{\varepsilon }}_{V}$ is the volume strain rate, $\bar{\sigma }$ is the effective stress, *F_i_* is the surface traction, *u_i_* is the velocity, *S* is the surface, *V* is the volume of a sample and *K* is the penalty constant (*K* = 10^6^).

Consider that a heat transfer can be conducted by solving the following equation [4]:

(2) $$k\nabla^{2}T+\dot{q}-\rho c\dot{T}=0,$$

where *k* is the thermal conductivity, *T* is the temperature, *ρ* is the specific density, *c* is the specific heat and $\dot{q}$ is the heat generation. The first term $k\nabla^{2}T$ and the third term $\rho c\dot{T}$ denote the heat-transfer rate and the heat-generation rate, respectively. The heat generation rate in the deformed body is induced by plastic deformation and is given as:

(3) $$\dot{q}=\alpha \bar{\sigma }\dot{\bar{\varepsilon }},$$

where *α* means the part of the mechanical energy converted into heat (assumed to be 0.9).

The equation, Equation (2), can be rewritten as follows using the weighted residual method:

(4) $$\int_{V}kT_{,i}\delta T_{,i}dV+\int_{V}\rho c\dot{T}\delta TdV-\int_{V}\alpha \bar{\sigma }\dot{\bar{\varepsilon }}\delta TdV-\int_{S_{q}}q_{n}\delta TdS=0\;,$$

where *q_n_* denotes the heat flux across the boundary surface. The temperature distribution of the workpiece can be received by solving Equation (4).

The effect of friction at the workpiece–tool interface can be allowed for by the following equation [1,19]:

(5) $$f=-mk\left[\frac{2}{\pi }arctg\left(\frac{\left|u_{s}\right|}{u_{0}}\right)\right]\frac{u_{s}}{\left|u_{s}\right|},$$

where *m* is the friction factor, *k* is the shear yield stress, *u_s_* is the velocity vector of the workpiece relative to the anvil and *u*_0_ is a very small positive number compared to |*u_s_*|.

### 2.2. Investigated Material and Simulation Procedures

**Figure 1:**
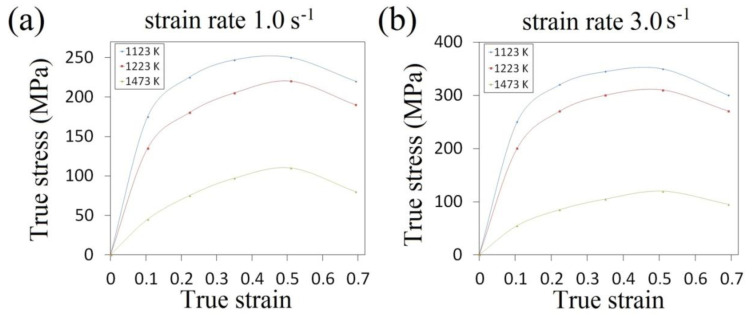
The relationships between true stress and true strain for X32CrMoV12-28: (**a**) strain rate 1.0 s^−1^; (**b**) strain rate 3.0 s^−1^.

For the analysis of the cogging process, a finite element method-based commercial software SFTC DEFORM-3D V.11 program was applied. It renders it possible to conduct thermal-mechanical simulation of the forging processes in a three-dimensional strain state. For obtaining the properties of the investigated X32CrMoV12-28 die steel at elevated temperatures, experimental plastometric tests were conducted; these included the compression of axial-symmetrical samples which had the following dimensions: ø30 × 30 mm on a cam plastometer (MAEKAWA-Japan), taking into consideration the different values of strain, strain rates and the determined scope of the temperature of hot deformation $\left(\bar{\sigma }=\bar{\sigma }\left(\bar{\varepsilon },\dot{\bar{\varepsilon }},T\right)\right)$. The samples were deformed within the following scope of temperatures: 1123–1473 K; within the following scope of true strain: 0.105–0.693; and within the following scope of strain rates: 0.20–3.0 s^−1^. The course of the relationships between true stress and true strain and temperature for two selected strain rates—1.0 and 3.0 s^−1^—and also for three temperatures—1123, 1223 and 1473 K—are presented in Figure 1. The determined curves $\bar{\sigma }=f\left(\bar{\varepsilon }\right)$ demonstrate the characteristic maximum of true stress, which is observed in the case of true strain amounting to approximately 0.50, whereas the situation of this maximum is not dependent upon temperature.

**Figure 2:**
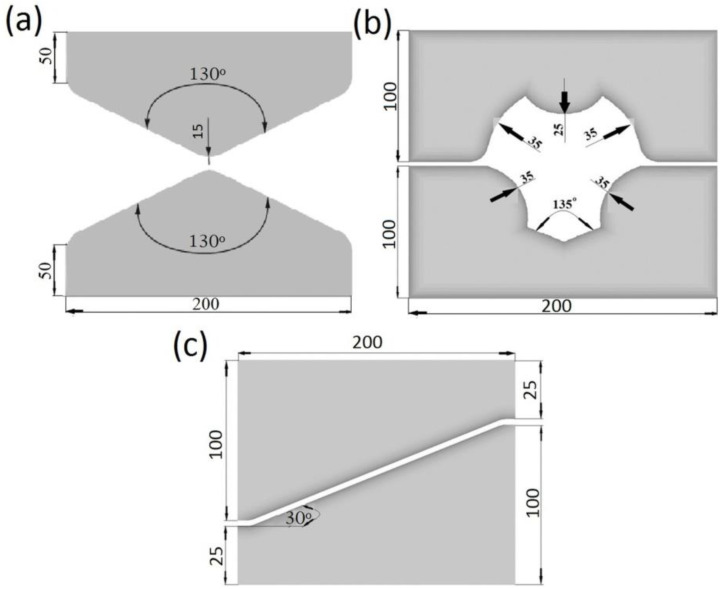
Shape of anvils: (**a**) convex; (**b**) assembly of three-radial; (**c**) with skew working surfaces.

In numerical calculations, the charge ø80 mm × 200 mm was adopted; the calculations were conducted for X32CrMoV12-28 die steel, which had the following chemical composition: C—0.35%, Mn—0.45%, Cr—3.50%, Si—0.40%, V—0.50% and Mo—2.50. Forging was conducted in several consecutive technological passes with rotating by an angle of 90°, with maintaining the constant true reduction ratio *ε_h_*= 0.35 and the constant feed rate amounting to *l_w_* = 0.75 in every pass. Forging was conducted in two anvils for preparing—one convex, which had the impression angle of 130° (Figure 2a), and one in a three-radial assembly (Figure 2b)—and also in an anvil with a skew working surface (Figure 2c) for conducting a complete technological process.

**Figure 3:**
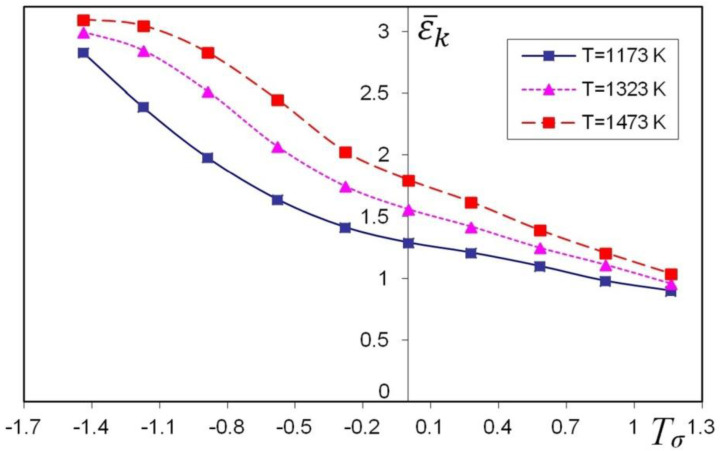
Relationship between the threshold effective strain and stress triaxiality and temperature for the X32CrMoV12-28 die steel.

For the X32CrMoV12-28 die steel, experimental model sample upsetting was conducted in order to determine the relationships between the threshold strain and the stress triaxiality and temperature. The calculations were made for samples with heigh/-diameter ratio H/D = 0.40–1.50 for different values of the friction factor, 0.05–0.70, and for three temperatures, 1173, 1323 and 1473 K, by the finite element method. The initial diameter was ø30.0 mm and the initial height of the sample was determined from the adopted height/diameter ratio value (0.40, 0.50, 0.75, 1.0, 1.25 and 1.50). The samples were deformed to a final height at which cracks were observed on the free surface. The values of the friction factor used in the calculations were also determined on the basic of additional ring compression tests. The determined component of the stress tensor *σ_z_* on the free surface, in the middle of the sample height, assumed negative values in all cases (compressive stress). Tensile stress *σ_Θ_* acted in the circumferential direction; hence, the calculated mean stresses on the free surface, in the middle of the upset samples, assumed variable values from tensile to compression. The conducted research allowed to establish the relationships between the threshold effective strain and stress triaxiality and temperature for the X32CrMoV12-28 die steel, which are presented in Figure 3.

The process conditions in the FE simulation and some thermal physical properties of the workpiece were as follows: (1) the friction factor between the billet and tool was 0.7 (the friction factor was determined on the basis of additional ring compression tests); (2) the reduction ratio varied from 0.01 to 0.35 at intervals of 0.01, the assumed deformation value of 0.35 resulted from experimental analyses of forging the tested steel under production conditions (the reduction ratio is defined as lnh_1_/h, where h and h_1_ are the height of the initial and deformed billet, respectively); (3) the feed rate amounted to 0.75 in every pass [8,25]; (4) the environment temperature was 293 K; (5) the initial temperature of forging was 1373 K (it resulted from the performed plastometric tests); (6) the initial temperature of the anvils was 573 K (i.e., the anvil heating-up temperature under industrial conditions); (7) the pressing speed of anvils was 20 mm/s [33]; (8) the convection coefficient to environment was 0.02 N/(s mm °C) [27,32,33]; (9) the heat transfer coefficient between the deformed material and anvils was 5 N/(s mm °C) [34].

### 2.3. Ductile Fracture Criteria

A clear majority of failure criteria developed in the literature are based upon the values of stresses integrated by means of material strain. The obtained critical values of particular criteria in the situations of the formation of cracks are dependent upon the kind of process, the geometry of samples and the shape of applied tools [33,34,35].

The basic form of the modified strain criterion of the loss of cohesion can be expressed as:

(6) $$\Psi_{S}=\int_{0}^{{\bar{\varepsilon }}_{f}}\frac{d\bar{\varepsilon }}{{\bar{\varepsilon }}_{k}\left(T_{\sigma }\right)}\leq 1,$$

where ${\bar{\varepsilon }}_{k}\left(T_{\sigma }\right)$ is the function of workability threshold, $T_{\sigma }=\frac{\sigma_{m}}{\bar{\sigma }}$ is the stress triaxiality, $\bar{\varepsilon }$ is the effective strain, $\bar{\sigma }$ is the effective stress, ${\bar{\varepsilon }}_{f}$ is the effective strain of fracture and *σ_m_* is the mean stress.

The procedure of determining the area of potential cracking for the deformation criterion of the loss of cohesion adopts the assumption that in each and every calculation step ‘i’, and also for each and every element ‘j’, the following parameters will be calculated: the values of increments in effective strain $\Delta {\bar{\varepsilon }}_{ij}$, the values of the factor of the stress triaxiality and the values of the threshold strain matching the values of the factor of the stress triaxiality at a given calculation step ${\bar{\varepsilon }}_{k}\left(T_{\sigma }\right)$.

The deformation damage factor for the first pass (in N calculation steps) was determined with the application of the following dependence:

(7) $$\Psi_{s}=\sum_{i=1}^{N}\frac{\Delta {\bar{\varepsilon }}_{ij}}{{\bar{\varepsilon }}_{k}{\left(T_{\sigma }\right)}_{ij}}\leq 1,$$

Calculated with the application of the above-mentioned dependence (7), this factor is a non-dimensional parameter, and the critical value of it amounts to 1. The areas which have the critical value of the factor constitute situations where it is probable that microcracks will appear. For the cyclical cogging process conducted in two consecutive passes with rotating of the billet, the deformation damage factor was determined with the application of the following dependence:

(8) $$\Psi_{s}=\sum_{1}^{N}{\left[\frac{{\left({\bar{\varepsilon }}_{ij}\right)}_{1}+{\left(\Delta {\bar{\varepsilon }}_{ij}\right)}_{2}}{{\bar{\varepsilon }}_{k}{\left(T_{\sigma }\right)}_{ij}}\right]}^{t}\leq 1,$$

where *t* is microcrack healing coefficient.

Simultaneously, with initiating and increase in cracks in the course of plastic shaping, there occurs the process of ‘healing’ microcracks and slowing down their development. The process of bonding cracks in the conditions of triaxial compression also occurs as a result of their relative resituation. At high deformation temperatures, the microcrack ‘healing’ process also occurs as a result of the recrystallisation and diffusion processes. The microcrack ‘healing’ coefficient for a given material is the function of the factor of the stress triaxiality, effective strain rate and temperature ($t=f\left(T_{\sigma },\dot{\bar{\varepsilon }},T\right)$). Determining the precise value of the coefficient *t* is a complicated task, the objective of which is to investigate a particular steel in changeable thermal-mechanical conditions. The *t* coefficient can be determined at the moment of the crack’s appearance using Equation (8), based on the assumption that the critical value deformation damage factor *Ψ_s_* = 1.0. The calculations assume the constant of the *t* coefficient in every pass. The unknown value of the index exponent in the polynomial Equation (8) was determined by the method of successive approximations, using the developed computer program. For the investigated die steel, the determined value of coefficient *t* amounted to 1.485.

## 3. Results and Discussion

**Figure 4:**
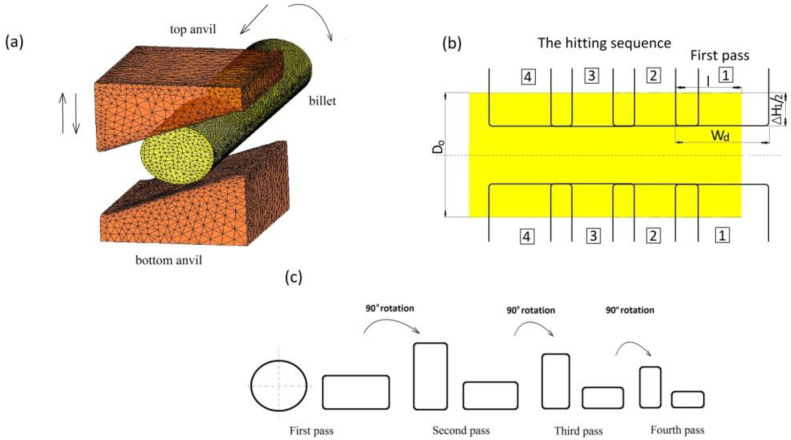
Schematic illustration of the cogging process: (**a**) finite element model for the forging experiment; (**b**) the hitting sequence for the cogging process (*l*/*w_d_* = 0.70; *l*/*D*_0_ = 0.75); (**c**) pass sequence for the cogging process.

In this paper, the distributions of effective strain, effective stress, mean stresses and temperature in the deformed X32CrMoV12-28 die steel in the conditions of hot cogging in three selected (original) forging tools are analyzed. The prediction of cracks in a forged material on the basis of the proposed deformation criterion of loss of cohesion was formulated. A schematic illustration of conducting particular technological passes and also the geometrical model of the anvils and the deformed material are presented in Figure 4. The forging process ought to be treated as a series of cyclically-repeating technological passes, and for that very reason, for convex anvils, two passes were applied, and the forging process in each pass was conducted in two stages: the first stage consisted in deformed in convex anvils, whereas the second stage consisted in eliminating formed concavities in flat anvils. Forging in assembly of three-radial anvils was conducted in two consecutive passes with the application rotating of the billet, whereas forging in anvils with skew working surfaces was conducted in the traditional manner, in four consecutive technological passes with the rotation of the deformed material by 90° (Figure 4c).

**Figure 5:**
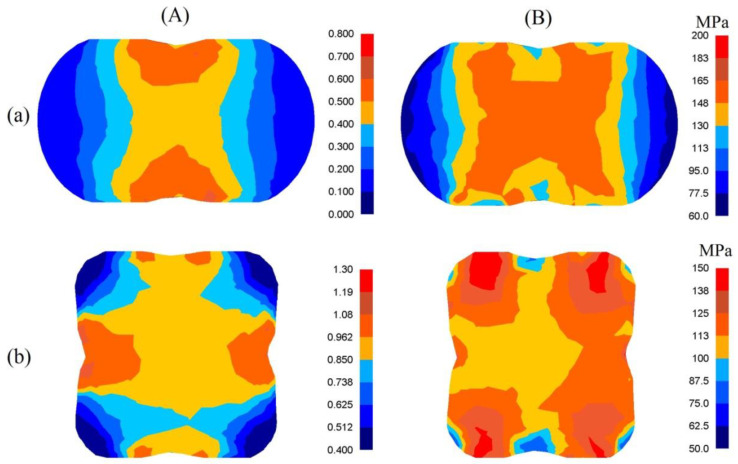
Distribution of effective strain (**A**) and effective stress (**B**) after the cogging process in convex anvils with an angle of 130°: (**a**) after first pass; (**b**) after the second pass (reduction ratio 0.70).

In Figure 5, the distributions of effective strain and effective stress after the cogging process in convex anvils with an angle of 130° after the first pass (Figure 5a), and after the second one (Figure 5b), are presented. The specific character of the forging process in convex anvils brings about the situation in which local strains on the cross-section of a deformed material after the first pass were varied but ensured, simultaneously, a good plastic deformation on the contacting surfaces. In the area adjacent to the surfaces of anvils, significant values of effective strain, exceeding the value of the set strain $(\bar{\varepsilon }$ = 0.686) two times, were observed. The lateral zones of a forging were the areas with smaller values of effective strain ($\bar{\varepsilon }$ = 0.114–0.229). The characteristic of the distributions of the effective stress, which are presented in Figure 5B-a, was similar. The application of the second technological pass brought about a significant increase in the uniformity of the distribution of effective strain. The areas with the highest values of effective strain included the central and lateral parts of the deformation valley, and these values amounted to $\bar{\varepsilon }\;$ = 0.962–1.07 (Figure 5A-b). It was solely insignificant areas, situated in the corners of a forging, that obtained the values of effective strain comparable with the value of the set strain ($\bar{\varepsilon }$ = 0.625–0.737). The uniformity of the distribution of effective strain was accompanied by the uniformity of distribution of the effective stress at the cross-section surface of a deformed material of a forging, and it amounted to $\bar{\sigma }$ = 110 MPa (Figure 5B-b).

**Figure 6:**
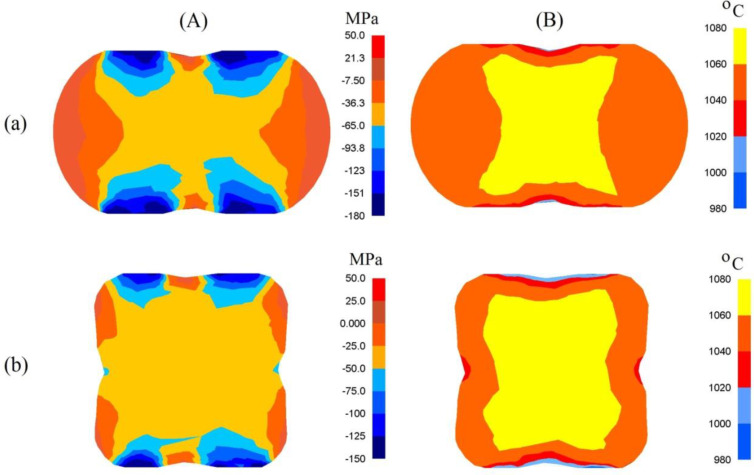
Distribution of mean stresses (**A**) and temperature (**B**) after the cogging process in convex anvils with an angle of 130°: (**a**) after first pass; (**b**) after the second pass (reduction ratio 0.70).

The presented distribution of the mean stresses in Figure 6A-a gives rise to a conclusive claim that on the lateral surface of deformed samples, after the first pass, tensile stresses may be observed. The highest values of compressing mean stresses are observed under the surfaces of convex anvils. After the second technological pass, virtually on the entire cross-section of a forging, favorable compressing stresses were observed; it is solely on the insignificant areas, situated in the corners, of a forging that insignificant values of tensile stresses may be observed (Figure 6A-b). A high value of effective strain on a clear majority of the parts of the surface of a cross-section of a forging shaped in convex anvils is conducive to generating significant values of the conversion of plastic deformation work into heat, which is conducive to thermal stability in the course of deformation in the central zones of a forging (Figure 6B). It was solely on the contact surfaces between hot metal and cooler anvils that a decrease in temperature, amounting to Δ*T* = 90–120 °C, was observed.

**Figure 7:**
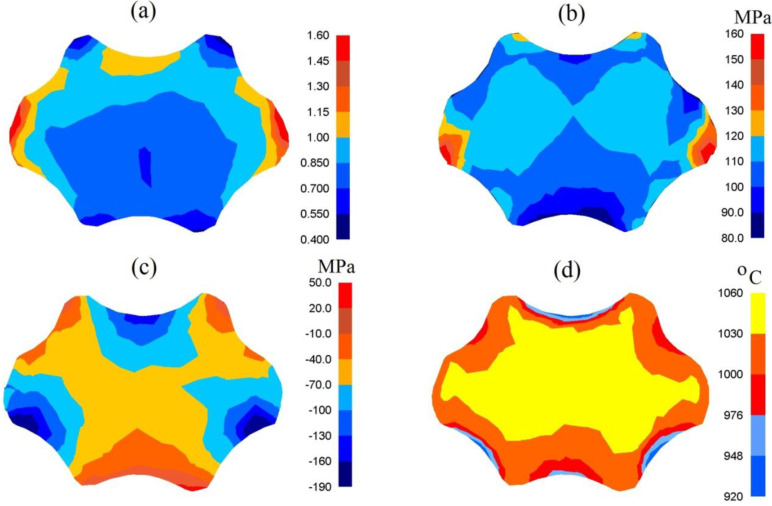
Distribution of the effective strain (**a**), effective stress (**b**), mean stresses (**c**) and temperature (**d**) after the cogging process in the assembly of three-radial anvils after the second pass (reduction ratio 0.70).

In Figure 7, the distribution of effective strain, effective stress, mean stresses and temperature after forging the samples of the X32CrMoV12-28 die steel in assembly of three-radial anvils after the second pass (*ε_h_* = 0.70) are presented. The anvils applied in investigations demonstrated a favorable influence upon the distribution of effective strain and effective stress in the cogging process. The highest values of effective strain were observed in the areas of a forging situated under the convex surfaces of anvils ($\bar{\varepsilon }$ = 1.00, Figure 7a). The central parts of a forging were the area with the moderately smaller values of effective strain $(\bar{\varepsilon }$ = 0.85). The advantage of forging in these anvils was a high uniformity of the distribution of effective strain. Even in the corners of a forging, not remaining under a direct impact exerted by anvils, the high value of effective strain, amounting to $\bar{\varepsilon }$ = 0.70–0.85, was obtained. The distribution of the effective stress was similar (Figure 7b). A large contact surface between a deformed material and a tool was the reason for a favorable triaxial compression in the central parts of a forging (Figure 7c). The presented distribution of mean stresses indicated the possibility of observing tensile stresses solely in very insignificant areas, not remaining under a direct impact exerted by anvils. The forging process was accompanied by a stable distribution of temperature in the central parts of a forging, brought about by the emission heat of plastic deformation work (Figure 7d). It was solely on the contact surface between a deformed material and anvils that a greater decrease in temperature, amounting to Δ*T* = 80–120 °C, was observed.

In the practice of forging, general-purpose flat anvils are applied most frequently; in them, forging a high-alloy steel is particularly non-favorable. Forging a round cross-section material on flat anvils in each and every case is connected with a high non-uniformity of the state of strain and also non-favorable local tensile stresses, determined, in addition to that, by the adopted values of feed rate and the reduction ratio. For that very reason, in this paper, improved flat anvils with skew working surfaces were applied, which rendered it possible to perform a complete the technological process without the time-consuming replacement of tools.

**Figure 8:**
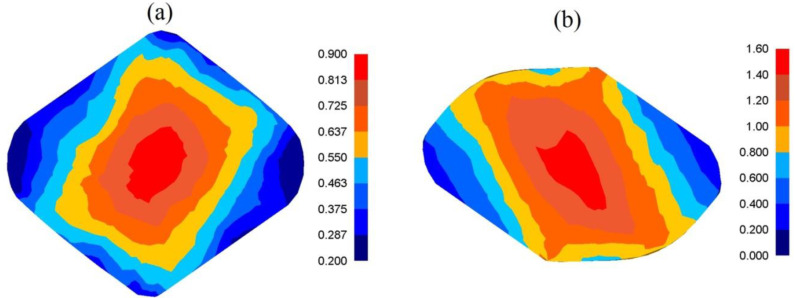
Distribution of the effective strain after the cogging process in anvils with skew working surfaces: (**a**) after the second pass (reduction ratio 0.70); (**b**) after the fourth pass (reduction ratio 1.40)**.**

In Figure 8, the distributions of effective strain after cogging the samples of the X32CrMoV12-28 die steel in anvils with skew working surfaces after the second technological pass (Figure 8a), and after the fourth one (Figure 8b), are presented. After the second pass, the areas with the highest values of effective strain included a significant central part of the deformation valley, and the values of them amounted to $\bar{\varepsilon }$ = 0.60–0.80 (Figure 8a). In the corners and on contact surfaces between a forging and a tool, the distribution of effective strain was moderately smaller, and values amounting to $\bar{\varepsilon }$ = 0.40–0.50 were obtained. After the fourth technological pass, a significant increase in the effective strain of the central zone of the deformation valley occurred; it amounted to $\bar{\varepsilon }$ = 1.40–1.60. The areas adjacent to the surface of anvils, and also the lateral zones of a forging, were areas which had significantly smaller values of effective strain (Figure 8b).

**Figure 9:**
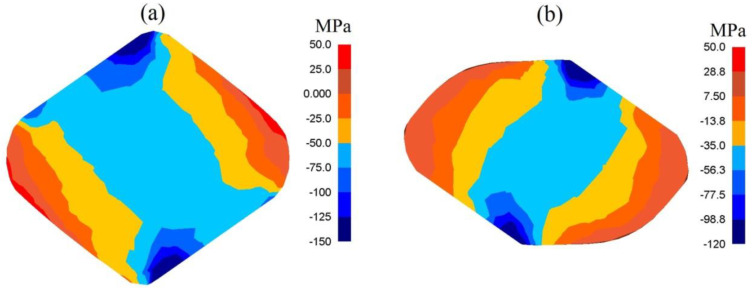
Distribution of mean stresses after the cogging process in anvils with skew working surfaces: (**a**) after the second pass (reduction ratio 0.70); (**b**) after the fourth pass (reduction ratio 1.40).

In Figure 9, the distributions of mean stresses after the cogging process in anvils with skew working surfaces after the second technological pass (Figure 9a), and after the fourth one (Figure 9b), are presented. Mean stresses *σ_m_* in the central parts of the deformation valley were compressing, which exerted a significant influence upon the internal quality of the forgings. It was solely in the lateral zones of a forging that it was possible to expect the presence of insignificant tensile stresses.

**Figure 10:**
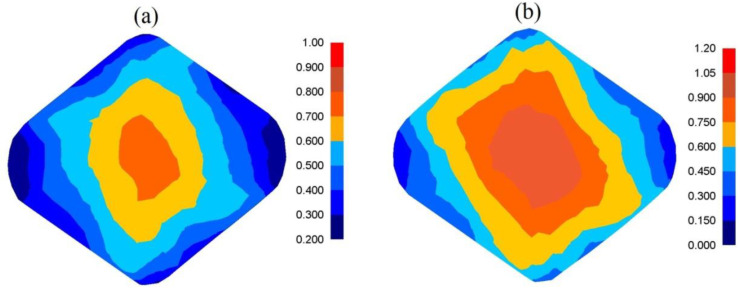
Distribution of effective strain after the cogging process in anvils with skew working surfaces after the second pass (reduction ratio 0.70): (**a**) feed rate of 0.75; (**b**) feed rate of 1.20.

In Figure 10, the distribution of effective strain after the second pass in the case of forging the samples of the X32CrMoV12-28 die steel in anvils with skew working surfaces with a changeable value of feed rate are presented. The area with the highest values of effective strain included a significant part of the axial zone of deformation, whereas an increase in the values of feed rate from *l_w_* = 0.75 up to *l_w_* = 1.20 brought about an increase in effective strain from $\bar{\varepsilon }$ = 0.89 to $\bar{\varepsilon }$ = 1.05.

**Figure 11:**
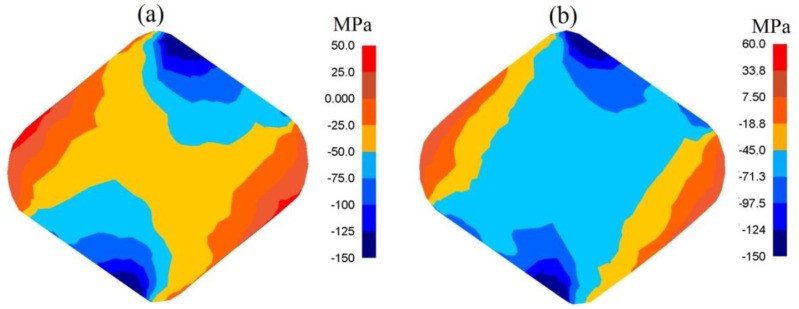
Distribution of mean stresses after the cogging process in anvils with skew working surfaces after the second pass (reduction ratio 0.70): (**a**) feed rate of 0.75; (**b**) feed rate of 1.20.

In Figure 11, the distribution of mean stresses after the second pass in the case of the samples of the X32CrMoV12-28 die steel in anvils with skew working surfaces with a changeable value of feed rate, are presented. For the analyzed scope of feed rate, mean stresses *σ_m_* in the central parts of the deformation valley were compressing. It is solely in the lateral zones of a forging that insignificant tensile stresses may be observed.

**Figure 12:**
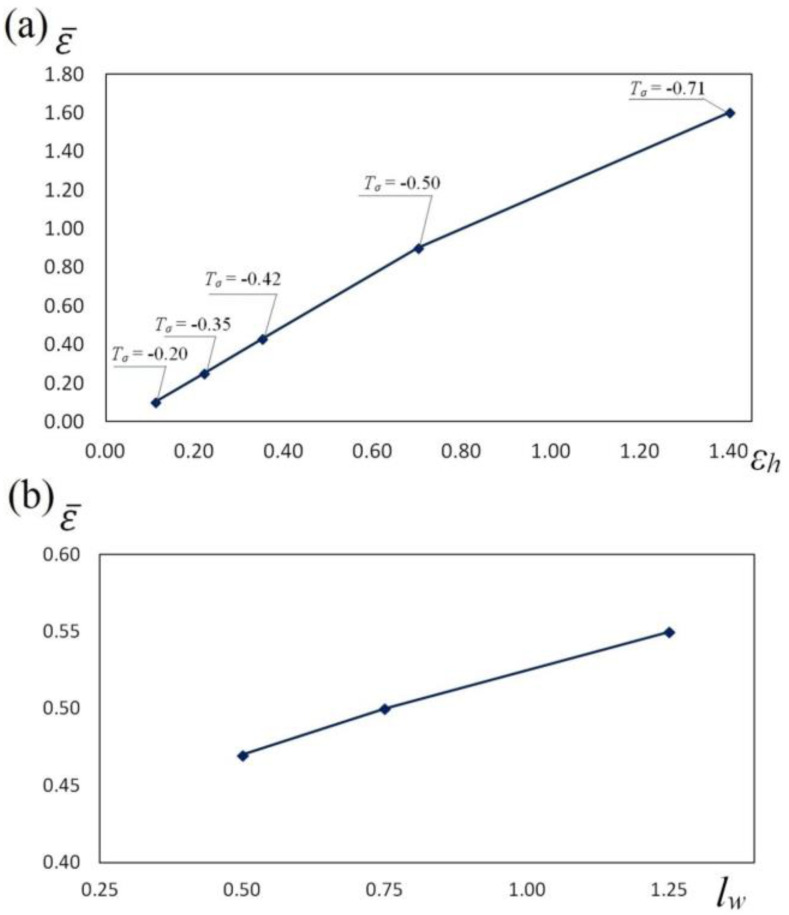
The influence of reduction ratio (**a**) and feed rate (**b**) on the effective strain $\bar{\varepsilon }$ during the cogging process of the X32CrMoV12-28 die steel specimens in anvils with skew working surfaces: (**a**) feed rate of 0.75; (**b**) reduction ratio of 0.70.

In Figure 12, the influence exerted by reduction ratio (a) and feed rate (b) upon the value of effective strain after cogging the samples of the X32CrMoV12-28 die steel in anvils with skew working surfaces is presented. The course of change in effective strain $\bar{\varepsilon }$ midway through the length of a sample in the function of total true strain (Figure 12a) and feed rate (Figure 12b) demonstrates that it is possible, in a planned and deliberate manner, to exert an influence upon an increase in the values of local effective strain in the determined zones of a forging, in the case of maintaining favorable values of the factor of stress triaxiality *T_σ_*_,_ significant for forging technologies.

**Figure 13:**
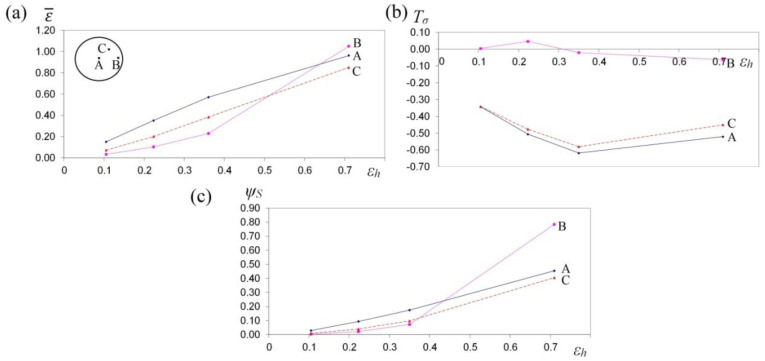
The variation in effective strain $\bar{\varepsilon }$ (**a**), stress triaxiality *T_σ_* (**b**) and the deformation damage factor *Ψ_S_* (**c**) at selected points A, B and C during the cogging process of the X32CrMoV12-28 die steel specimens in convex anvils with an angle of 130°.

**Figure 14:**
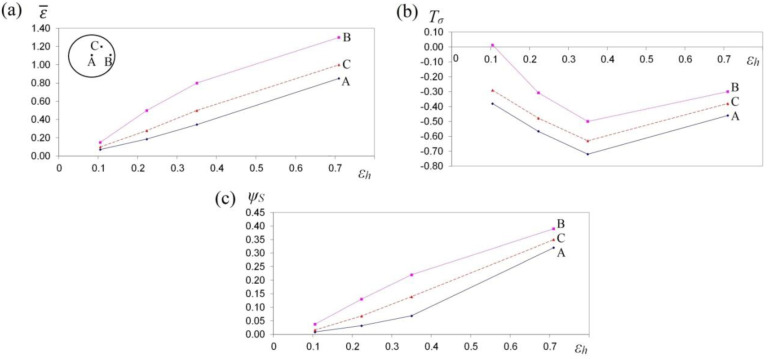
The variation in effective strain $\bar{\varepsilon }$ (**a**), stress triaxiality *T_σ_* (**b**) and the deformation damage factor *Ψ_S_* (**c**) at selected points A, B and C during the cogging process of the X32CrMoV12-28 die steel specimens in the assembly of three-radial anvils.

**Figure 15:**
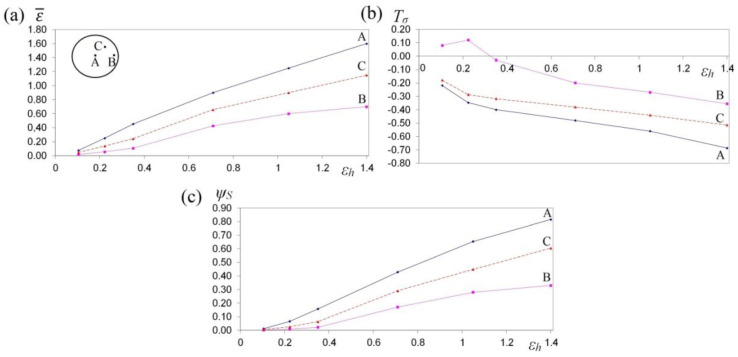
The variation in effective strain $\bar{\varepsilon }$ (**a**), stress triaxiality *T_σ_* (**b**) and the deformation damage factor *Ψ_S_* (**c**) at selected points A, B and C during the cogging process of X32CrMoV12-28 die steel specimens in anvils with skew working surfaces.

The collation of the course of changes in effective strain $\bar{\varepsilon }$, stress triaxality *T_σ_* and the deformation damage factor *Ψ_S_* in the function of reduction ratio in the course of the cogging process in investigated anvils is presented in Figure 13, Figure 14 and Figure 15. It was demonstrated that a reduction ratio exerted a significant influence upon the values of effective strain at particular points in the deformation valley. The specific character of the preparatory forging process in convex anvils, and in the three-radial ones, brought about moderately varied local strains on the cross-section of a deformed material; however, maximum plastic deformations were obtained simultaneously in a limited workability zone. The application of interoperational rotating of the billet and repeating deformation brought about a significant increase in the uniformity of effective strain in a cogging. For that very reason, preparatory forging in convex anvils and in assembly of three-radial anvils ensured intensive and uniform plastic deformation (Figure 13a and Figure 14a).

Large strains penetrated the central parts of a forging, where the values of effective strain significantly exceeded the value of the set strains, which exerted a positive influence upon deformation in the axial zone and was conducive to eliminating the discontinuities of metallurgical origin. In the center of the deformation valley (point A) in both tools, comparable values of effective strain were obtained, whereas in the lateral areas of a forging deformed in assembly of three-radial anvils (point B, Figure 14a), the highest values of effective strain, virtually two times exceeding the value of the set strain ($\bar{\varepsilon }$ = 1.30), were obtained. In Figure 15a, the course of changes in effective strain in the function of reduction ratio at the selected points of the cross-section in the course of cogging in anvils with skew working surfaces is presented. These anvils are general-purpose ones, and they render it possible to conduct a complete technological process, or they may be applied for the continuation of the technological process originated in convex anvils or in the assembly of three-radial ones. Large strains penetrated the central parts of a forging (point A). After the second technological pass, the values of effective strain significantly exceeded the value of the set strain ($\bar{\varepsilon }\;$ = 0.90), which exerted a positive influence upon deformation in the axial zone. At the remaining points of the cross-section, values of effective strain lower than the set strain were obtained. Nevertheless, forging at the subsequent technological passes with the application of interoperational rotating of the billet brought about a further significant increase in effective strain in the particular areas of the deformation valley, simultaneously contributing to an increase in the uniformity of the distribution of effective strain. After the fourth pass (reduction ratio 1.40) in the central zone of a forging, the values of effective strain $\bar{\varepsilon \;}$ = 1.40–1.60 were obtained.

In Figure 13b, Figure 14b and Figure 15b, the courses of changes in the stress triaxiality *T_σ_*, in the function of reduction ratio at the selected points of the cross-section in the course of forging a sample of the X32CrMoV12-28 die steel in three investigated anvils, are presented. The convex shape of the working surfaces of convex anvils, and of the assembly of three-radial ones, brought about the concentration of significant compression stresses in the central parts of the deformation valley, on insignificantly small areas, not significant for forging technologies, subjected to the impact exerted by tensile stresses. The stress triaxiality, accompanied by an increase in reduction ratio, particularly for applied anvils, adopted favorable negative values.

When there occurred an increase in reduction ratio, dangerous tensile stress situated in the lateral zones of a forging was no longer observed, and at reduction ratio *ε_h_* = 0.15–0.35, it was transforming entirely into compressing stress, whereas the intensity of these changes was determined, in addition to that, by the shape of the applied anvils. The most favorable conditions for reforging the particular zones of a forging were obtained in assembly of three-radial anvils, where, at a reduction ratio of 0.15 in the lateral zones of a forging, a favorable change from tensile stresses into the compressing ones was obtained (Figure 14b).

Determining the threshold workability of a material in forging processes is an important issue from the point of view of designing the best possible technologies of these processes. The results of the conducted investigations demonstrate a significant influence exerted by shape tools, and also by the values of technological parameters, upon the threshold value of effective strain, after exceeding which the disturbance of the cohesion of a material and ductile cracking of it occurs.

In Figure 13c, Figure 14c and Figure 15c, the courses of changes in the deformation damage factor *Ψ_S_* in the function of reduction ratio for three characteristic points—the center (A), lateral surface (B) and the internal point (C)—in the course of forging a sample of X32CrMoV12-28 die steel in three investigated anvils are presented. The presented values demonstrate significant differences in the values of deformation damage factor for the analyzed areas of the cross-section of a forging. The analysis of the results of the investigations, after the first pass (reduction ratio of 0.35), failed to demonstrate significant differences in the values of factor *Ψ_S_* in particular investigated tools. The scope of the values of deformation damage factor for the analyzed points of the cross-section amounted to *Ψ_S_* = 0.05–0.13. For samples deformed in convex anvils and anvils with skew working surfaces, the highest values of the deformation damage factor were observed in the central parts of a forging (point A). On the lateral surfaces (point B), lower values of the deformation damage factor were observed. For samples deformed in assembly of three-radial anvils, the highest values of the deformation damage factor were observed in the lateral zones of a forging (point B). In the central parts of a forging, the lowest values of the deformation damage factor (*Ψ_S_* = 0.05) were obtained. In the zone determined by point C, the mean values of factor *Ψ_S_* were observed. In the distributions of the deformation damage factor, it is possible to observe a close connection with a local distribution of the values of effective strain, accompanied by increase in reduction ratio; the varied local values of effective strain, and also the values of deformation damage factor, were more and more visible.

A two-fold increase in reduction ratio (*ε_h_* = 0.70) brought about an intensive increase in the values of the deformation damage factor, *Ψ_S_* = 0.30–0.35, in the particular zones of plastic deformation represented by points A, B and C, whereas the character of these changes for particular investigated anvils was similar to the results obtained after the first technological pass (*ε_h_* = 0.35). A further increase in the total reduction ratio up to the value of *ε_h_* = 1.40 (after the fourth technological pass) in the course of forging in anvils with skew working surfaces brought about a proportional increase in the deformation damage factor at particular points of the cross-section (Figure 15c). Nevertheless, reaching the threshold value of the deformation damage factor and the formation of discontinuities in the course of forging in anvils with skew working surfaces originated significantly later for the higher values of reduction ratio. The possibility of prediction of the ductile fracture of a shaped material during the cogging process is an indispensable element of designing the best possible technological process.

**Figure 16:**
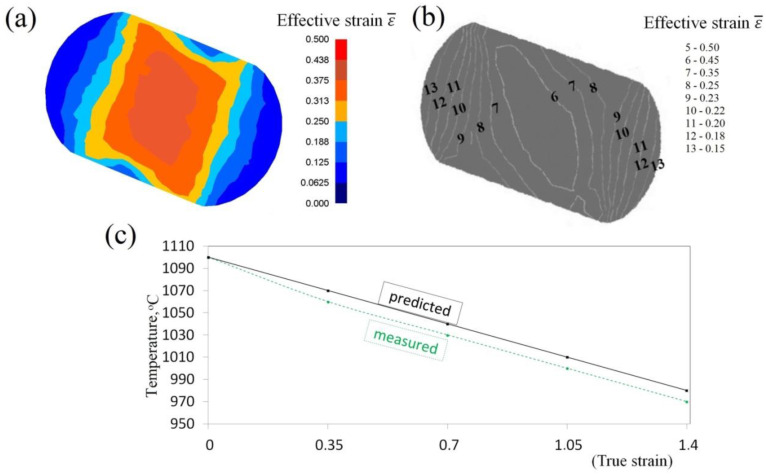
Comparison of theoretical and experimental results after the cogging process in anvils with skew working surfaces: theoretical (**a**) and experimental (**b**) distributions of effective strain (*l_w_* = 0.75, *ε_h_* = 0.35); (**c**) temperature on the surface of the specimens.

Experimental confirmation of the results of numerical investigations into the distribution of effective strain, and also temperature, after forging in anvils with skew working surfaces is presented in Figure 16. The state of strain was determined with the application of the coordination grid method. Experiments were conducted on cylindrical samples, φ80 × 150 mm, made of the X32CrMoV12-28 die steel, on which, in the parting line, a square coordination grid (3.0 × 3.0 mm) with the precision of 0.01 mm was plotted. The situation of nodes before and after deformation was measured with the accuracy of 0.01 mm on a test bench, provided with the toolmaker’s measuring microscope coupled with a terminal. The samples were deformed on a hydraulic press (exerting a pressure of 2500 kN). In order to determine the components of Cauchy strain tensors, the relocation field of a sixth-degree double-parameter polynomial was approximated, whereas coefficients were determined with the application of the method of least squares, taking into consideration the condition of a constant volume (Lagrange multiplier method). The measurement of temperature at selected points on the surface of a deformed cogging, after particular technological passes, was conducted with the application of a thermovision camera. For good results 10% deviation was accepted. The comparative analysis of effective strain, and also of temperature, based upon numerical calculations and obtained by means of experiments, demonstrated their being mutually commensurate to a relatively high degree.

## 4. Conclusions

The conducted numerical investigations, confirmed by means of experiments, rendered it possible to determine the local values characterizing the state of strain and stress and the distribution of temperature and also to predict ductile fracture in the form of the proposed deformation damage factor in the course of the cogging process for X32CrMoV12-28 die steel in convex anvils, in assembly of three-radial ones and anvils with skew working surfaces (not applied in this practice of forging). These three kinds of applied forging tools ought to be included into the group of highly-effective ones in the aspects of distribution of effective strain and stress, absence of tensile stresses in the axial zones of a forging and also significant thermal stability in the internal layers of a deformed material. Particularly favorable was the application, at the initial stages of forging, of a special assembly of three-radial anvils. In the course of forging in these anvils, no limited workability zones were observed. The maximum values of effective strain were found upon the surface of a forging, and insignificantly smaller values were found in the central parts of it, in the absence of non-favorable tensile stresses. In the surface zones of a forging, a lot of heat of plastic deformation work was generated, and for that very reason, the cogging process was accompanied by small changes in temperature in the volume of the deformation valley, contributing to a high uniformity of the distribution of effective strain.

The possibility of prediction of the ductile fracture of a shaped material in the cogging process is an indispensable element of designing the best possible forging technologies. The principal parameters changing the values of the deformation damage factor (*Ψ_S_*) in the course of cogging were the shape of anvils and the value of reduction ratio. The investigated changes in the deformation damage factor in the course of forging in different investigated anvils were the source of data on the values of factor *Ψ_S_* in particular zones of plastic deformation and rendered it possible to predict the situation and the phase of deformation in which the loss of cohesion of a deformed material will occur. At the initial stages of forging, it is proposed to apply highly effective convex anvils or assembly of three-radial ones; further, and also finally, forging may be conducted in universal anvils with skew working surfaces.
